# Altered Rbfox1-Vamp1 pathway and prefrontal cortical dysfunction in schizophrenia

**DOI:** 10.21203/rs.3.rs-2944372/v1

**Published:** 2023-06-01

**Authors:** Youjin Chung, Samuel Dienel, Matthew Belch, Kenneth Fish, George Ermentrout, David Lewis, Daniel Chung

**Affiliations:** University of Pittsburgh; University of Pittsburgh; University of Pittsburgh; University of Pittsburgh; University of Pittsburgh; University of Pittsburgh

## Abstract

Deficient gamma oscillations in prefrontal cortex (PFC) of individuals with schizophrenia appear to involve impaired inhibitory drive from parvalbumin-expressing interneurons (PVIs). Inhibitory drive from PVIs is regulated, in part, by RNA binding fox-1 homolog 1 (Rbfox1). Rbfox1 is spliced into nuclear or cytoplasmic isoforms, which regulate alternative splicing or stability of their target transcripts, respectively. One major target of cytoplasmic Rbfox1 is vesicle associated membrane protein 1 (Vamp1). Vamp1 mediates GABA release probability from PVIs, and the loss of Rbfox1 reduces Vamp1 levels which in turn impairs cortical inhibition. In this study, we investigated if the Rbfox1-Vamp1 pathway is altered in PVIs in PFC of individuals with schizophrenia by utilizing a novel strategy that combines multi-label in situ hybridization and immunohistochemistry. In the PFC of 20 matched pairs of schizophrenia and comparison subjects, cytoplasmic Rbfox1 protein levels were significantly lower in PVIs in schizophrenia and this deficit was not attributable to potential methodological confounds or schizophrenia-associated co-occurring factors. In a subset of this cohort, Vamp1 mRNA levels in PVIs were also significantly lower in schizophrenia and were predicted by lower cytoplasmic Rbfox1 protein levels across individual PVIs. To investigate the functional impact of Rbfox1-Vamp1 alterations in schizophrenia, we simulated the effect of lower GABA release probability from PVIs on gamma power in a computational model network of pyramidal neurons and PVIs. Our simulations showed that lower GABA release probability reduces gamma power by disrupting network synchrony while minimally affecting network activity. Finally, lower GABA release probability synergistically interacted with lower strength of inhibition from PVIs in schizophrenia to reduce gamma power non-linearly. Together, our findings suggest that the Rbfox1-Vamp1 pathway in PVIs is impaired in schizophrenia and that this alteration likely contributes to deficient PFC gamma power in the illness.

## Introduction

Impairments in cognitive processes, such as working memory, are among the core clinical features of schizophrenia^1,2^. Working memory is associated with synchronous neural activity at gamma frequency in the prefrontal cortex (PFC)^3,4^, and individuals with schizophrenia exhibit lower PFC gamma oscillation power during working memory tasks^5–7^. Thus, a critical step in developing novel therapeutics for schizophrenia involves identifying the mechanisms that impair the generation of gamma oscillations in the PFC.

Gamma oscillations require strong phasic inhibition from parvalbumin-expressing interneurons (PVIs) that synchronize the firing of excitatory pyramidal cells^8,9^. Multiple postmortem studies reported lower levels of molecular markers for inhibitory drive from PVIs in the PFC in schizophrenia^10–14^, and animal model studies showed that reducing inhibitory drive from these neurons lowers gamma power^15,16^. Thus, impaired inhibitory drive from PVIs is proposed to be a critical contributor to deficient PFC gamma oscillations in schizophrenia^17^.

Inhibitory synaptic drive from PVIs appears to be regulated, in part, by RNA binding fox-1 homolog 1(Rbfox1)^18,19^. Rbfox1 is one of the three Rbfox paralogues with an RNA recognition motif that binds to the conserved hexanucleotide, (U)GCAUG, within its target transcripts^20,21^. In mouse cortex, Rbfox1 is highly expressed in PVIs and its target transcripts are enriched in genes that regulate synaptic transmission^19,22,23^. Furthermore, the loss of Rbfox1 results in abnormal post-transcriptional regulation of its target transcripts and impaired cortical inhibition^18,19,24^. Finally, a recent study showed that that PFC levels of Rbfox1 mRNA are lower in schizophrenia^25^. These findings suggest that alterations in Rbfox1 levels could contribute to impaired inhibitory drive from PVIs by disrupting post-transcriptional regulation of its target transcripts. However, direct evidence for altered Rbfox1 expression in PVIs in schizophrenia, and dysregulated regulation of its target transcripts involved in impaired cortical inhibition, remain unknown.

Understanding how Rbfox1 could contribute to impaired cortical inhibition in schizophrenia requires investigating the levels of specific Rbfox1 protein isoforms, as its post-transcriptional effect on target transcripts differs based on the inclusion or exclusion of nuclear localization signal (NLS)^26^. For example, the Rbfox1 isoform that contains NLS localizes to the nucleus (nuclear Rbfox1) and binds to splicing loci of target transcripts to regulate their alternative splicing^26,27^, whereas the Rbfox1 isoform that lacks NLS localizes to the cytoplasm (cytoplasmic Rbfox1) and binds to 3’ UTR to enhance the stability of its target transcripts^18,22^. Recently, Vuong et al^18^ showed that one of the target transcripts of cytoplasmic Rbfox1 is vesicle associated membrane protein 1 (Vamp1), a key component of the SNARE complex involved in neurotransmitter release from presynaptic terminals^28,29^. They also found that 1) Vamp1 is highly enriched in PVIs and regulates the release probability of GABA neurotransmitters from these neurons; 2) 3’ UTR of Vamp1 is a direct target of cytoplasmic Rbfox1 and the loss of Rbfox1 reduces the stability of Vamp1 transcripts; and 3) the loss of Rbfox1 results in lower release probability of inhibitory drive from PVIs, which is rescued by re-expression of Vamp1. Together, these findings strongly suggest that cytoplasmic Rbfox1 is a critical regulator of inhibitory drive from PVIs via its effect on the transcriptional stability of Vamp1. Therefore, examining the levels of cytoplasmic Rbfox1 isoform and Vamp1 mRNA in PVIs could provide important insights into molecular mechanisms underlying impaired cortical inhibition and deficient PFC gamma oscillations in schizophrenia.

Here, we tested the hypothesis that schizophrenia is associated with lower levels of cytoplasmic isoform of Rbfox1 protein and its target transcript Vamp1 in prefrontal PVIs. To test this hypothesis, we applied a novel technical strategy that combines multi-label in situ hybridization and immunohistochemistry in postmortem human brain to quantify the levels of cytoplasmic Rbfox1 isoform and Vamp1 mRNA in the same PVIs in the PFC of individuals with schizophrenia. We then investigated the functional consequence of altered Rbfox1-Vamp1 pathway in schizophrenia by simulating how changing the release probability of inhibitory drive from PVIs affects gamma power in a computational model network. Our combined findings from postmortem studies and computational modeling suggest that alterations of the Rbfox1-Vamp1 pathway in PVIs are a critical component of pathogenic mechanisms underlying deficient PFC gamma oscillations in schizophrenia.

## Methods

### Human subjects

Brain specimens (n = 40) were obtained during routine autopsies conducted at the Office of the Allegheny County of Medical Examiner (Pittsburgh, PA) after consent was obtained from the next-of-kin. An independent team of clinicians made consensus DSM-IV diagnoses for each subject using the results of structured interviews with family members, review of medical records, neuropathology exam, and toxicology findings^30^. The same approach was used to confirm the absence of a psychiatric diagnosis in unaffected comparison subjects. All procedures were approved by the University of Pittsburgh Committee for Oversight of Research and Clinical Training Involving Decedents and the Institutional Review Board for Biomedical Research.

To reduce biological variance between subject groups, each subject with schizophrenia was matched to one unaffected comparison subject perfectly for sex and as closely as possible for age (**Supplementary Table 1**). The mean age, postmortem interval (PMI), tissue storage time, and RNA integrity number (RIN) did not differ between subject groups (Table 1). Brain pH significantly differed between subject groups (t_19_ = 2.2, p = 0.039), but the mean difference was 0.2 pH units and of uncertain biological relevance^31^. Twenty pairs of subjects were used for the multi-label fluorescent immunohistochemistry assay, and the three comparison subjects from this cohort were used to compare Rbfox1 levels across different interneuron subtypes. From the twenty pair cohort, ten pairs with the greatest within-pair differences in cytoplasmic Rbfox1 levels were selected for the multi-label *in situ* hybridization combined with immunohistochemistry assay. Three comparison subjects from this smaller cohort were used to assess Vamp1 mRNA levels across cortical layers and between PVIs and non-PV neurons.

### Non-human primates

Male, adult macaque monkeys (*Macaca fascicularis*; n = 3) were perfused transcardially with 1% paraformaldehyde followed by 4% paraformaldehyde in phosphate buffer^32^. Brains were removed and 5– 6mm thick coronal blocks were fixed in 4% paraformaldehyde at 4°C for 6 hours. Tissue blocks were immersed in increasing gradients of sucrose solutions and stored at −30°C until sectioned for immunohistochemistry. All housing and experimental procedure followed the guidelines of the US Department of Agriculture and NIH guidelines for the Care and Use of Laboratory Animals and were conducted with the approval of the University of Pittsburgh Institutional Animal Care and Use Committee.

### Multi-label fluorescent immunohistochemistry

Paraformaldehyde-fixed tissue blocks containing left PFC area 9 were sectioned coronally at 40μm on a cryostat. Sections were permeabilized with 0.3% Triton X-100 in PBS for 1 hour at room temperature, blocked using 20% donkey serum for 2 hours at room temperature, and then incubated in PBS containing primary antibodies for 72 hours at 4°C. Tissue sections were washed three times in PBS and then incubated in PBS containing secondary antibodies for 24 hours at 4°C. After washing three times in PBS, sections were stained with 4’,6-diamidino-2-phenylindole (DAPI; Invitrogen, Cat#D1306) diluted in 1:50,000 in PBS for 10 minutes at room temperature. Sections were then mounted on slides, cover- slipped, and coded to obscure subject number and diagnosis. The following primary antibodies were used in this study: mouse anti-Rbfox1 (1:1000; Millipore, Cat#MABE985), chicken anti-PV (1:1000; Synaptic System, Cat#195006), goat anti-calretinin (CR; 1:1000; Swant, Cat#CG1) and rabbit anti-calbindin (CB; 1:1000; Swant, Cat#CB38). Information on antibody validation is included in the Supplementary Methods.

### Multi-label fluorescent in situ hybridization combined with immunohistochemistry

Fresh-frozen tissue blocks containing right PFC area 9 were sectioned coronally at 20μm, thaw-mounted onto SuperFrost slides (Thermo Fisher, Cat#12–550-15), and stored at − 80°C until processed for multi-label fluorescent *in situ* hybridization (RNAscope) combined with immunohistochemistry (see **Supplementary Methods** for details). In short, sections were fixed in phosphate-buffered 4% paraformaldehyde at 4°C for 15 minutes, dehydrated through a series of ethanol washes, and incubated with 3% hydrogen peroxide at room temperature for 10 minutes. Sections were then incubated in a mixture of RNAscope probes for Vamp1 (Advanced Cell Diagnostics [ACD], Cat#1046151-C1 tag) and PV (ACD, Cat#422181-C3 tag) mRNAs at 40°C for 2 hours. Amplification and detection steps were performed using the RNAscope Multiplex Fluorescent Detection Kit v2 (ACD, Cat#323110). Sections were then incubated with amplification probes Amp1, Amp2, and Amp3 sequentially, followed by horseradish peroxidase (HRP) enzymes targeting each channel (HRP-C1 and HRP-C3) at 40°C for 15 minutes. Following the labeling of Vamp1-C1 and PV-C3 probes with Opal 570 and 690 (Akoya Biosciences, Cat#OP-001003; OP001006), respectively, sections were incubated in mouse anti-Rbfox1 antibody (1:200; Millipore, Cat#MABE985) at 4°C overnight, labeled with Opal 520 (Akoya Biosciences, Cat#OP-001001) at 40°C for 15 minutes, and counterstained with DAPI at room temperature for 30 seconds.

### Image acquisition and post image processing

**Figure 1:**
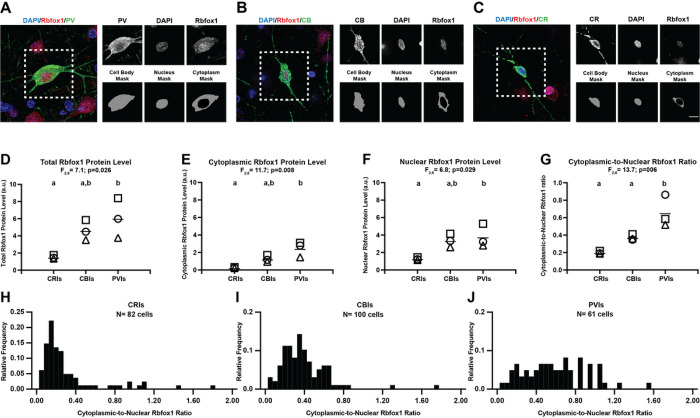
Cytoplasmic Rbfox1 isoform is enriched in PVIs in human PFC. (A-C) Left: Colored panels are representative images of paraformaldehyde-fixed human PFC immunolabeled with antibodies against PV (A), CB (B) or CR (C) and Rbfox1 and counterstained with DAPI. Right: Upper panel shows the single images of each label from the boxed area. Lower panel shows masking of the cell body for each interneuron subtype using PV, CB or CR labeling and the nucleus using DAPI staining. The nucleus mask is subtracted from the cell body mask to generate the cytoplasm mask. Scale bar= 10μm. (D-G) Plots comparing the levels of total Rbfox1 (D), cytoplasmic Rbfox1 (E) and nuclear Rbfox1 (F), and the C/N ratio of Rbfox1 isoform (G) across CRIs, CBIs and PVIs. Each symbol represents one unaffected comparison subject and each bar indicates the mean value. Results of one-way ANOVA analyses are shown above the graph. Interneuron subtypes not sharing the same letters are significantly different (p<0.05 after Tukey’s multiple comparison). (H-J) Frequency distributions (bars) of C/N Rbfox1 ratio across CRIs (H), CBIs (I) and PVIs (J). Greater C/N Rbfox1 ratio (G) and the right shift in the frequency distribution in PVIs (J) relative to CBIs and CRIs (H, I) suggest that cytoplasmic Rbfox1 isoform is enriched in PVIs relative to other major interneuron subtypes in human PFC.

Images from fluorescent immunohistochemistry assay were acquired on an Olympus IX81 microscope with a spinning disk confocal unit and Hamamatsu EM-CCD digital camera using a 60X objective. Interneurons were sampled from layers 2 and 4, defined as 10–20% and 50–60% of the pia-to-white matter distance, respectively^33^. A stack of 20 images of 512×512 pixels and a step size of 0.25μm was selected from each section using a previously published method for systematic sampling^30^. Fluorescent channels were deconvolved using Autoquant’s Blind Deconvolution algorithm to improve the image contrast by reducing out-of-focus fluorescence. For each sampled neuron, the z-plane containing the largest surface area of its cell body and two z-planes above and below that plane were selected for masking segmentation. A total of 8,485 z-planes were manually masked for this process. Masking of PV, CR, or CB immunoreactivity was used to define the cell body of each type of interneuron whereas the masking of DAPI staining was used to define the nucleus of each interneuron. The nuclear mask was then subtracted from the cell body mask to determine the borders of the cytoplasmic mask for a given neuron (Fig. 1A–C). Previous studies have shown that levels of Rbfox1 in the cytoplasmic or the nuclear compartment predominantly reflect the levels of the corresponding Rbfox1 isoform^19,22,26,34^. Therefore, we measured the mean intensities of Rbfox1 immunoreactivity within the cell body, nuclear and cytoplasmic masks to assess the levels of total Rbfox1, nuclear Rbfox1, and cytoplasmic Rbfox1, respectively.

Images from the combined fluorescent *in situ* hybridization and immunohistochemistry assay were acquired on a wide-field Olympus IX83 microscope equipped with a Hamamatsu ORCA-FLASH 4.0 sCMOS camera using a 60X objective. PVIs were sampled from layer 4 as defined above. A stack of 75 images of 1024×1024 pixels and a step size of .25μm was selected from each section. For each image, the DAPI and Rbfox1 channels were deconvolved using the Autoquant’s Blind Deconvolution algorithm and an average 2D projection over the z-axis was created. PV and Vamp1 mRNA grains were masked using the previously described iterative segmentation method (see **Supplementary Methods**)^30,35^. Rbfox1 protein and DAPI labeling were manually masked to define the cell body and the nucleus, respectively, of Rbfox1-immunoreactive neurons. The nuclear mask was then subtracted from the cell body mask to produce the cytoplasmic mask for each Rbfox1-immunoreactive neuron (Fig. 4C). A naïve Bayes model^36^ was used to classify all sampled Rbfox1-immunoreactive neurons as PVIs or non-PV neurons based on the density of PV mRNA grains each neuron contained. Finally, the density of Vamp1 mRNA grains and the mean intensity of Rbfox1 immunoreactivity within the cytoplasmic mask of individual PVIs were quantified.

### Statistical analysis

To assess the effect of interneuron subtypes on dependent measures in human and monkey immunohistochemical experiments, one-way analysis of variance (ANOVA) with Tukey’s multiple comparison test was used. To assess the main effect of diagnosis on the dependent measures in the studies of schizophrenia and unaffected comparison subjects, two analysis of covariance (ANCOVA) models were used. The paired ANCOVA model included protein or mRNA level as the dependent variable, diagnostic group as the main effect, subject pair as a blocking factor, and brain pH, PMI, RIN (mRNA only), and tissue storage time as covariates. This model accounts for the matching by sex and age and for the parallel tissue processing of sections from subject pairs but is not a true statistical paired design. Thus, we also used an unpaired ANCOVA model which included protein or mRNA level as the dependent variable and age, sex, brain pH, PMI, RIN (mRNA only), and tissue storage time as covariates. The paired and unpaired analyses from the ANCOVA showed comparable levels of statistical significance for all dependent variables. The final models only included the covariates that achieved significance in the initial model. Analyses from the paired ANCOVA are reported in the Results, and analyses from both paired and unpaired models are included in the figures.

The potential influence of co-occurring factors that are frequently present in schizophrenia subjects (e.g., prominent mood symptoms (i.e., schizoaffective disorder), substance abuse or dependence at the time of death; use of nicotine, antidepressants, benzodiazepines, and/or valproic acid at the time of death; or death by suicide) was assessed by ANCOVAs with each factor as a main effect and sex, age, brain pH, PMI, storage time and RIN (mRNA only) as covariates.

### Computational model network of pyramidal neurons and PVIs

To simulate the effect of GABA release probability on gamma power, we built a computational model network that consisted of 80 regular-spiking pyramidal neurons and 20 fast-spiking PVIs using the quadratic integrate-and-fire model as previously described^17^ (See **Supplementary Methods** or details). Cells were connected to all other cells in the network (i.e., all-to-all connection). Each excitatory synapse contained AMPA and NMDA conductance and each inhibitory synapse contained GABA conductance. Parameters used to model the spiking property and the synaptic conductance of pyramidal and PVIs are described in the Supplementary Methods. Release probability was introduced to inhibitory synapses by applying the Bernoulli process to the synaptic gating rule, so that postsynaptic receptors in pyramidal neurons were activated by presynaptic spikes in PVIs at a given probability. External excitatory currents were applied to pyramidal neurons to initiate network activity. For each simulation trial, power spectral density was taken on the sum of all excitatory synaptic currents into pyramidal neurons to compute peak gamma power. Results of each experiment are the average of 200 trials.

## Results

### Cytoplasmic Rbfox1 isoform is enriched in PVIs in human PFC.

To investigate if cytoplasmic Rbfox1 isoform levels are altered in PVIs in schizophrenia, we first examined whether Rbfox1 and its isoforms are enriched in these neurons in human PFC. To assess the enrichment of Rbfox1, we compared the levels of Rbfox1 protein across PVIs, calbindin-expressing interneurons (CBIs), and calretinin-expressing interneurons (CRIs), the three major interneuron subtypes in human PFC^37^ (Fig. 1A–C). Total Rbfox1 protein levels, defined as the mean level of Rbfox1 immunoreactivity in the entire cell body of each interneuron, significantly differed (F_2_,6=7.1, p = 0.026) across interneuron subtypes (Fig. 1D). Total Rbfox1 protein levels were 4.0-fold greater in PVIs and 3.0-fold greater in CBIs relative to CRIs. Post hoc analyses revealed that total Rbfox1 protein levels were significantly greater in PVIs than in CRIs but did not differ between CBIs and CRIs. These findings are unlikely to reflect any interneuron differences in susceptibility of Rbfox1 immunoreactivity to postmortem effects as a similar pattern of Rbfox1 protein levels across three interneuron types was also present in the PFC of perfused macaque monkeys (**Supplementary Fig. 1**).

Next, we examined whether the protein levels of cytoplasmic and nuclear Rbfox1 isoforms in PVIs differ from CBIs or CRIs. Cytoplasmic Rbfox1 isoform levels, defined as mean levels of Rbfox1 immunoreactivity in the cytoplasm, significantly differed (F_2_,6=11.7, p = 0.008) across interneuron subtypes (Fig. 1E). Cytoplasmic Rbfox1 levels were 9.6-fold greater in PVIs and 5.0-fold greater in CBIs relative to CRIs. Post hoc analyses revealed that cytoplasmic Rbfox1 protein levels were significantly greater in PVIs relative to CRIs but did not differ between CBIs and CRIs. Similarly, nuclear Rbfox1 isoform levels, defined as mean levels of Rbfox1 immunoreactivity in the nucleus, significantly differed (F_2_,6=6.8, p = 0.029) across interneuron subtypes (Fig. 1F). Nuclear Rbfox1 isoform levels were 3.0-fold greater in PVIs and 2.6-fold greater in CBIs relative to CRIs. Post hoc analyses revealed that nuclear Rbfox1 isoform levels were significantly greater in PVIs relative to CRIs but did not differ between CBIs and CRIs.

To compare the magnitude of the shift in splicing between cytoplasmic and nuclear isoforms of Rbfox1 across interneuron subtypes, we assessed the cytoplasmic-to-nuclear (C/N) ratio of Rbfox1 isoform levels. The C/N ratio of Rbfox1 isoform levels significantly differed (F_2_,6=13.7, p = 0.006) across interneuron subtypes (Fig. 1G). The Rbfox1 C/N ratio was 1.8-fold and 3.3-fold greater in PVIs relative to CBIs and CRIs, respectively. Post hoc analyses revealed that Rbfox1 C/N ratio was significantly greater in PVIs relative to both CBIs and CRIs. Consistent with this finding, the distribution of the C/N ratio across individual interneurons was shifted to the right in PVIs relative to both CBIs and CRIs. (Fig. 1H–J). These findings suggest that Rbfox1 is highly expressed in PVIs in human PFC and that the splicing of Rbfox1 is shifted to produce relatively more cytoplasmic than nuclear isoform in PVIs compared to other major interneuron subtypes.

### Cytoplasmic Rbfox1 protein levels in PVIs are lower in schizophrenia.

**Figure 2:**
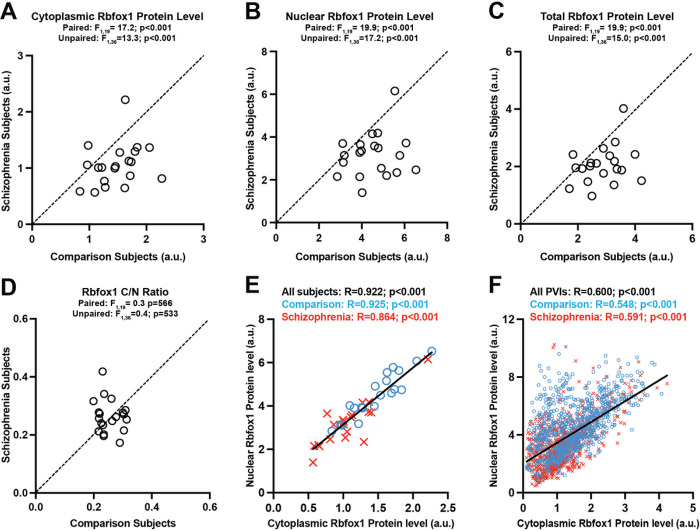
Protein levels of cytoplasmic Rbfox1 in PVIs are lower in PFC of schizophrenia. Rbfox1 in PVIs are lower in PFC of schizophrenia. (A-D) Scatter plots for mean protein levels of cytoplasmic Rbfox1 (A), nuclear Rbfox1 (B), total Rbfox1 (C) and the C/N Rbfox1 ratio (D) in PVIs for each unaffected comparison subject (x-axis) and schizophrenia subject (y-axis) in a pair. Data points below the diagonal unity line indicate a lower level in schizophrenia relative to matched unaffected comparison subjects. Results of paired and unpaired ANCOVA analyses are shown above each plot. Protein levels of Rbfox1 isoforms in PVIs are all lower in schizophrenia without evidence of shift in the relative abundance of each isoform. (E, F) Correlation graphs plotting cytoplasmic Rbfox1 levels on x-axis and nuclear Rbfox1 levels on y-axis across subjects (E) and across individual PVIs sampled from comparison (N=890 cells) and schizophrenia (N=807 cells) subjects (F). Positive correlations between levels of cytoplasmic and nuclear Rbfox1 suggest their expression levels are co-regulated.

To investigate whether schizophrenia is associated with altered levels of cytoplasmic Rbfox1 in PVIs, we quantified the protein levels of cytoplasmic Rbfox1 in PVIs from the PFC of 20 pairs of schizophrenia and unaffected comparison subjects. Mean levels of cytoplasmic Rbfox1 immunoreactivity were significantly (F_1_,19=17.2, p < 0.001) 29% lower in PVIs from individuals with schizophrenia relative to unaffected comparison subjects (Fig. 2A). In addition to lower levels of cytoplasmic Rbfox1, the mean levels of nuclear Rbfox1 (F1,19=19.9, p < 0.001) and total Rbfox1 (F1,19=19.9, p < 0.001) immunoreactivities were also lower in PVIs in schizophrenia subjects (Fig. 2B, C). However, the Rbfox1 C/N ratio did not significantly differ between subject groups (Fig. 2D). Moreover, the levels of cytoplasmic and nuclear Rbfox1 isoforms were significantly positively correlated across both unaffected comparison and schizophrenia subjects (Fig. 2E) and these correlations were also evident across individual PVIs (Fig. 2F). These findings suggest that the levels of cytoplasmic and nuclear Rbfox1 isoforms are co-regulated and that schizophrenia is associated with lower levels of both isoforms in PVIs without evidence of shift in splicing.

**Figure 3:**
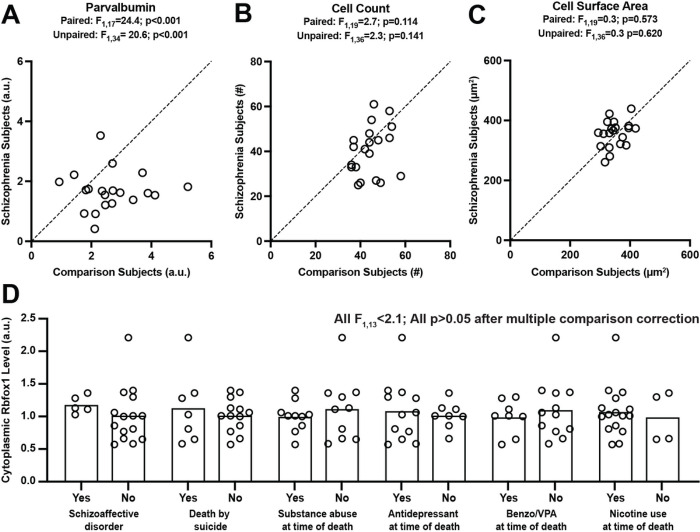
Lower cytoplasmic Rbfox1 levels in PVIs in schizophrenia are not due to methodological confounds or schizophrenia-associated co-occurring factors. (A-C) Scatter plots for mean PV protein levels (A), mean number of sampled PVIs (B) and mean surface area of PVIs for each unaffected comparison subject (x-axis) and schizophrenia subject (y-axis) in a pair. Lower PV protein levels in PVIs in schizophrenia without significant differences in the number of sampled neurons suggest that our sampling method robustly captured PVIs that are affected in the illness. Similar surface area of PVIs between two subject groups demonstrates the lack of disease effect on the masking of PVIs. (D) Bar graphs showing the effect of each co-occurring factors on mean protein levels of cytoplasmic Rbfox1 levels in PVIs in schizophrenia. Circles represent the levels of dependent measures for individual subject. For each co-occurring factor, schizophrenia subjects are grouped by the absence (No) or presence (Yes) of the factor listed on the x- axis. All F values<2.1 and all p values>0.05 after multiple comparison correction, suggesting lower cytoplasmic Rbfox1 levels in schizophrenia are not due to disease-associated co-occurring factors.

Next, we investigated whether lower cytoplasmic Rbfox1 levels in PVIs in schizophrenia might be due to methodological confounds or diagnosis-associated co-occurring factors. Consistent with our prior study using the sample subject cohort^30^, the mean level of PV immunoreactivity was significantly (F1,17=24.4, p < 0.001) 37% lower in schizophrenia (Fig. 3A), validating that our sampling method robustly captured PVIs that are affected in the illness. The number and the surface area of sampled PVIs did not differ between subject groups (Fig. 3B, C), suggesting that our findings are not due to methodological confounds associated with sampling or masking of PVIs, respectively. Finally, the levels of cytoplasmic (Fig. 3D), nuclear or total Rbfox1 (**Supplementary Fig. 2**) did not significantly differ as a function of a diagnosis of schizoaffective disorder; the presence of a substance use disorder at time of death; use of nicotine, antidepressants, benzodiazepines, or valproic acid at the time of death; or death by suicide. Together, these findings support our hypothesis that schizophrenia is associated with lower cytoplasmic Rbfox1 levels in PVIs and suggest that this alteration is not due to methodological confounds or diagnosis-associated co-occurring factors.

### Lower cytoplasmic Rbfox1 protein levels predict lower mRNA levels of Vamp1 in PVIs.

**Figure 4:**
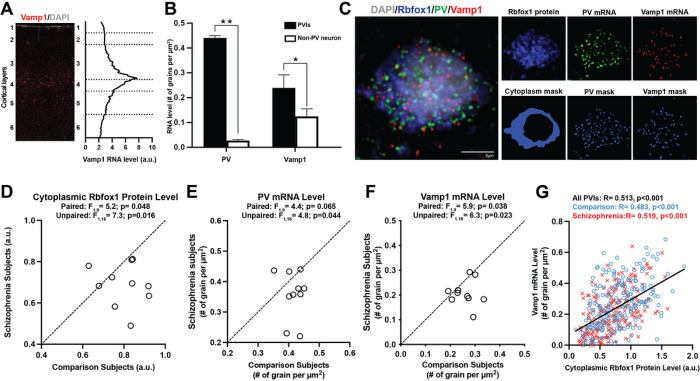
Lower cytoplasmic Rbfox1 protein level predicts lower Vamp1 mRNA level in PVIs. (A) Left: Representative image of a fresh-frozen human PFC labeled with riboprobe for Vamp1, a major target transcript of cytoplasmic Rbfox1, counterstained with DAPI. Right: Mean Vamp1 mRNA fluorescent intensity as a function of cortical layers in human PFC (N=3). (B) Bar graphs comparing the density of PV (left) and Vamp1 (right) mRNA grains in PVIs and non-PV neurons sampled in human PFC (N=3; total of 64 PVIs and 188 non-PV neurons were sampled). **p<0.001 and *p<0.05 from the Student’s t test. (C) Left: Representative image of a fresh-frozen human PFC labeled with an antibody against Rbfox1, riboprobes for PV and Vamp1 mRNAs, and counterstained with DAPI. Scale bar= 10 m. Right: Upper panel shows the single images of Rbfox1 protein, PV mRNA grains and Vamp1 mRNA grains from the combined image. Lower panel shows the masking of cytoplasm used to quantify cytoplasmic Rbfox1 and the masking of PV and Vamp1 mRNA grains. (D-F) Scatter plots for mean cytoplasmic Rbfox1 protein levels (D) and the mean density of PV (E) and Vamp1 (F) mRNA grains in PVIs for each unaffected comparison subject (x-axis) and schizophrenia subject (y-axis) in a pair. (G) Correlation graph plotting cytoplasmic Rbfox1 protein level on x-axis and the density of Vamp1 mRNA grain on y-axis across PVIs sampled from comparison (N=214 cells) and schizophrenia (N=198 cells) subjects. Positive correlation between these two measures suggests that lower Vamp1 mRNA levels in PVIs could be due to lower cytoplasmic Rbfox1 protein levels in schizophrenia.

Cytoplasmic Rbfox1 has been shown to enhance the mRNA stability of its major target Vamp1^18^. To investigate if lower cytoplasmic Rbfox1 protein levels predict lower Vamp1 mRNA levels in PVIs in schizophrenia, we first explored whether Vamp1 is highly expressed in these neurons in human PFC. Vamp1 mRNA levels were highest in layer 4 of human PFC where PVIs are most abundant^37^ (Fig. 4A). In this layer, the density of Vamp1 mRNA grains was two-fold greater in PVIs relative to non-PV neurons (Fig. 4B), consistent with previous mouse studies reporting that Vamp1 is enriched in PVIs^18,38^.

Next, we explored if we could co-detect Vamp1 mRNA and Rbfox1 protein in the same PVIs in human PFC. Using a novel approach that combines multi-label *in situ* hybridization and immunohistochemistry^39^, we were able to detect the presence of Rbfox1 immunoreactivity and Vamp1 mRNA grains in the same PVIs in fresh-frozen human PFC sections (Fig. 4C). To validate that this approach can reliably sample affected PVIs in fresh-frozen PFC sections of schizophrenia, we selected the 10 subject pairs with the largest difference in cytoplasmic Rbfox1 protein levels in fixed PFC sections. Then, we quantified cytoplasmic Rbfox1 protein levels and the density of PV mRNA grains in PVIs in fresh-frozen PFC sections from these 10 pairs. The mean levels of cytoplasmic Rbfox1 immunoreactivity (F1,9=5.2, P = 0.048; Fig. 4D) and the mean density of PV mRNA grains (F1,9=4.4, P = 0.065; Fig. 4E) were each 14% lower in PVIs in schizophrenia, respectively. These findings demonstrate that our approach robustly detects deficits in both mRNA and protein levels in affected PVIs in schizophrenia using fresh- frozen PFC sections.

Finally, we investigated the relationship between the levels of cytoplasmic Rbfox1 protein and Vamp1 mRNA in PVIs in schizophrenia. The mean density of Vamp1 mRNA grains was significantly (F_1_,9=5.9, P = 0.038) 20% lower in PVIs in PFC of schizophrenia relative to comparison subjects (Fig. 4F). Furthermore, the levels of cytoplasmic Rbfox1 immunoreactivity were significantly positively correlated with the density of Vamp mRNA grains across individual PVIs in both unaffected comparison and schizophrenia subjects (Fig. 4G). Together, these findings demonstrate that Vamp1 mRNA levels are lower in PVIs in schizophrenia and suggest that this alteration could be due to deficits in cytoplasmic Rbfox1 levels in these neurons.

### Lower release probability of GABA from PVIs reduces gamma power in a computational model network.

**Figure 5:**
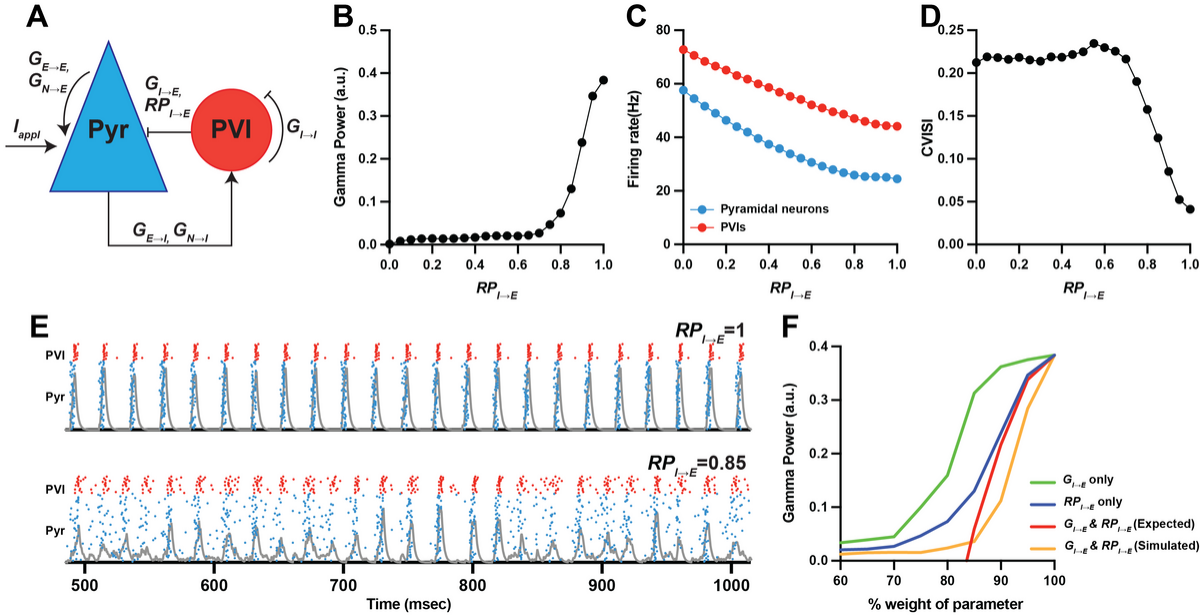
Lower release probability of GABA from PVIs reduces gamma power in a computational model network. (A) Schematic diagram of the model network illustrating connectivity between pyramidal neurons (Pyr) and PVIs. GE→E and GN→E indicate AMPA and NMDA conductance in recurrent excitatory inputs to pyramidal cells, respectively. GE→I and GN→I indicate AMPA and NMDA conductance in excitatory inputs to PVIs, respectively. GI→E and GI→I indicate GABA conductance to pyramidal neurons and PVIs, respectively. RPI→E indicates release probability applied to GI→E to simulate changes in GABA release probability onto pyramidal neurons. Iappl is the external current applied to pyramidal neurons to initiate network activity. (B-D) Plot of RPI→E versus gamma power (B), firing rate (C) of pyramidal neurons (blue) or PVIs (red), and CVISI (D) computed as an average over 200 trials. From RPI→E=1 to 0.7, gamma power sharply decreases, accompanied by a steep increase in CVISI but without significant changes in the firing rate. (E) Representative raster plots (blue dots indicate pyramidal neurons, N=80; red dots indicate PVIs, N=20) and network activity (gray) over time for RPI→E=1 (above) and 0.85 (below). Relative to RPI→E=1, desynchronization (greater horizontal scatter of blue and red dots) is seen at RPI→E=0.85. (F) Plot of gamma power versus four different parameter conditions. GI→E (green) and RPI→E (blue) was each reduced from its value that produced maximal gamma power (corresponding to 100% parameter weight on x-axis). The magnitude of reduction in gamma power from independently lowering GI→E and RPI→E was summed to predict the magnitude of reduction by lowering two parameters together (red). The actual gamma power reduction simulated by lowering two parameters together (orange) is greater than the expected reduction (red) until gamma power reaches a stable nadir at 85% weight for each parameter, suggesting that lower GI→E and lower RPI→E synergistically interact to non-linearly reduce gamma power in the model network.

Vamp1 regulates the release probability of neurotransmitters and the loss of Vamp1 levels in PVIs reduces the release probability of GABA from these neurons^18,28,29^. To explore the functional consequence of Rbfox1-Vamp1 alterations in schizophrenia, we simulated the effect of reduced release probability of GABA from PVIs (RPI◊E) on gamma power in a model network of pyramidal neurons and PVIs (Fig. 5A).

Maximum gamma power occurred at RPI◊E=1 (Fig. 5B). Gamma power then sharply decreased with RPI◊E<1 and reached a stable nadir at RPI◊E≤0.7, demonstrating that reducing RPI◊E disrupts the generation of gamma oscillations in the model network. To further investigate the network properties affected by lower RPI◊E, we assessed its effect on network activity, measured by the firing rates of pyramidal and PVIs, and its effect on network synchrony, measured by the coefficient of variation of the interspike interval (CVISI) of pyramidal neurons. Decreasing RPI◊E from 1 to 0.7 had minimal effect on neuronal firing rates (Fig. 5C), but steeply increased CVISI (Fig. 5D). These findings suggest that lower RPI◊E reduces gamma power primarily by disrupting network synchrony (i.e., higher CVISI) while minimally affecting network activity (Fig. 5E).

In addition to lower release probability, the strength of PVI-mediated inhibition is also thought to be lower in schizophrenia based on prior studies reporting lower levels of the GABA synthesizing enzyme GAD67 in PVIs^10,40,41^ and lower levels of GABAA1 receptor α1 subunit (GABAA1α1) mRNA in pyramidal neurons^42,43^. To investigate how deficits in the release probability and the strength of inhibitory drive from PVIs together influence PFC gamma power in schizophrenia, we simulated the effect of the interaction between RPI◊E and PV-mediated inhibitory strength (GI◊E) on gamma power. To simulate the effect of this interaction, GI◊E (Fig. 5F; green line) or RPI◊E (Fig. 5F; blue line) was each reduced incrementally from its value that produced maximum gamma power (i.e., GI◊E=0.7 and RPI◊E=1, corresponding to 100% parameter weight in Fig. 5F). Next, the magnitude of the reduction in gamma power from each parameter was summed to predict the expected reduction in gamma power from lowering both parameters together (Fig. 5F; red line). These values were then compared to the actual gamma power reduction simulated by lowering GI◊E and RPI◊E concurrently (Fig. 5F; orange line). Our simulation showed that the actual magnitude of reduction in gamma power was greater than the expected reduction until GI◊E and RPI◊E were lowered by 15%, after which the magnitude of gamma power reached a stable nadir. Thus, these findings suggest that lower GI◊E and lower RPI◊E synergistically interact to reduce gamma power non-linearly in the model network.

## Discussion

Prior studies have suggested that alterations in the Rbfox1 pathway could be associated with schizophrenia. First, genome-wide association studies linked a locus containing the RBfox1 gene with an increased risk of schizophrenia^25,44^. Second, large scale RNA-seq studies identified Rbfox1 as a hub gene for a set of co-expressed transcripts that are enriched for schizophrenia risk genes^45^ or downregulated in the illness^46^. Third, levels of Rbfox1 mRNA were reported to be lower in schizophrenia^25^. However, the nature of alterations in Rbfox1 protein isoforms and their target transcripts, and their potential impact on PFC dysfunction, had not been investigated in schizophrenia. In this study, we report that schizophrenia is associated with lower protein levels of cytoplasmic Rbfox1 isoform in PVIs in PFC of schizophrenia. This finding was due to an overall reduction in total Rbfox1 protein levels and not due to a shift in the ratio of Rbfox1 isoforms. Also, this finding was not confounded by our sampling or masking of PVIs.

Furthermore, none of the assessed schizophrenia-associated co-occurring factors accounted for this alteration. Finally, the levels of Vamp1 mRNA, a major target transcript of cytoplasmic Rbfox1, were lower in PVIs in schizophrenia and were predicted by lower cytoplasmic Rbfox1 protein levels across these neurons. Together, these findings support the hypothesis that the nature of Rbfox1 pathway alterations in schizophrenia includes lower levels of cytoplasmic Rbfox1 protein isoform and of its target Vamp1 mRNA in prefrontal PVIs.

Cytoplasmic Rbfox1 is hypothesized to regulate the expression of its target transcripts by competing with the binding of microRNAs in 3’ UTR^22^. In mouse Vamp1 3’UTR, the 3’-most Rbfox binding site is located immediately adjacent to a microRNA response element (MRE) for miRNA-9, and the binding of cytoplasmic Rbfox1 has been shown to protect Vamp1 mRNA from the repressive effect of miRNA-9^18^. The MRE for miRNA-9 and the adjacent Rbfox1 binding site are also present in human Vamp1 3’UTR^47^, suggesting that the regulatory role of cytoplasmic Rbfox1 on Vamp1 mRNA levels is conserved in humans. Consistent with this idea, our study shows a positive correlation between the protein levels of cytoplasmic Rbfox1 and the mRNA levels of Vamp1 in PVIs, which are both lower in schizophrenia. In concert, these findings support the regulatory role of cytoplasmic Rbfox1 on Vamp1 levels in human PFC and suggest that lower cytoplasmic Rbfox1 protein levels contribute to lower Vamp1 mRNA levels in PVIs in schizophrenia.

Alternative splicing of Rbfox1 between cytoplasmic and nuclear isoforms is regulated by neuronal activity^26^. However, we found similar reductions in the levels of total and both Rbfox1 isoforms in PVIs in schizophrenia, as well as lower levels of PV which is expressed in an activity-dependent fashion^48,49^. These findings suggest that lower cytoplasmic Rbfox1 in schizophrenia is not due to activity-dependent shifts in alternative splicing and suggest that some factor(s) other than lower excitatory drive to PVIs^30^ accounts for lower Rbfox1 levels in the illness.

Alterations in the Rbfox1-Vamp1 pathway in PVIs are thought to impair cortical inhibition by reducing the release probability of GABA^18^. To investigate the effect of lower GABA release probability in schizophrenia, we simulated its effect on the generation of gamma oscillations in a computational model network. Our simulation showed that lower GABA release probability can robustly reduce gamma power predominantly by disrupting the synchrony of pyramidal neuron firing. Prior studies have shown that lower GABA release probability can also arise from alterations in ErbB4^50^, DISC1^51^ or C4^52^ pathway, each of which has been shown to be disrupted in schizophrenia^53–56^. Thus, deficits in multiple molecular mechanisms, including Rbfox1-Vamp1 alterations, could result in reduced GABA release probability that contributes to impaired generation of PFC gamma oscillations in schizophrenia.

In addition to lower release probability, lower strength of inhibitory synapses from PVIs is also thought to be associated with schizophrenia given prior studies demonstrating lower levels of GAD67 in PVIs^10,40,41^ and GABAA1α1 in pyramidal neurons^42,43^ in the illness. Our simulation suggests that molecular pathways regulating release probability and those that regulate the strength of inhibitory synapses may synergistically influence the generation of PFC gamma oscillations. Thus, these findings support the idea that lower PFC gamma power in schizophrenia emerges from non-linear interactions among alterations in multiple synaptic parameters that mediate cortical inhibition^17^.

Our study is limited in assessing the effect of antipsychotics on Rbfox1 levels since all but one of the schizophrenia subjects used in this study were on antipsychotics at the time of death. However, in published gene expression data from the PFC of subjects with bipolar disorder, transcript levels of Rbfox1 did not differ between individuals on or off antipsychotic medications at the time of death^57^. Moreover, transcript levels of Rbfox1 did not differ in monkeys treated with haloperidol or clozapine for 6 months relative to comparison monkeys^58^. Thus, the effect of antipsychotics is unlikely to account for lower Rbfox1 levels in schizophrenia.

In conclusion, our study demonstrates that schizophrenia is associated with lower levels of cytoplasmic Rbfox1 isoform in prefrontal PVIs which could account for lower Vamp1 mRNA levels in the same neurons. Furthermore, our simulations predict that these alterations in the Rbfox1-Vamp1 pathway in PVIs are critical components of pathogenic mechanisms underlying deficient PFC gamma oscillation power in schizophrenia. As multiple target transcripts of Rbfox1 have been shown to regulate synaptic transmission^18,19,22–24^, future studies investigating alterations in additional Rbfox1 target transcripts and their involvement in PFC dysfunction may further validate the role of Rbfox1 in schizophrenia.

## Figures and Tables

**Table 1:** Summary characteristics of human subjects used in this study.

	Unaffected comparison subjects	Schizophrenia subjects	
**Sex**	15M/5F	15M/5F	
**Race**	16W/4B	14W/6B	
	**Mean/SD**	**Mean/SD**	**Paired t-test**
**Age (years)**	46.3/12.1	45.2/11.8	t_19_ = 1.3, p = 0.209
**Brain pH**	6.7/0.2	6.5/0.3	t_19_ = 2.2, p = 0.039
**PMI (hours)**	16.4/5.5	15.4/6.3	t_19_ = 0.7, p = 0.470
**RIN**	8.1/0.51	8.1/0.52	t_19_ = 0.2, p = 0.873
**Storage time (months)**	110.8/33.9	103.5/28.3	t_19_ = 0.9, p = 0.335
